# Evolving Definitions of Mental Illness and Wellness

**Published:** 2009-12-15

**Authors:** Ronald W. Manderscheid, Carol D. Ryff, Elsie J. Freeman, Lela R. McKnight-Eily, Satvinder Dhingra, Tara W. Strine

**Affiliations:** Mental Health and Substance Use Programs, Global Health Sector, SRA Intl, Inc. Dr Manderscheid is also affiliated with the Bloomberg School of Public Health, Johns Hopkins University, Rockville, Maryland.; University of Wisconsin-Madison, Madison, Wisconsin; Maine Department of Health and Human Services, Augusta, Maine; Centers for Disease Control and Prevention, Atlanta, Georgia; Centers for Disease Control and Prevention, Atlanta, Georgia; Centers for Disease Control and Prevention, Atlanta, Georgia

## Abstract

Understanding of the definitions of wellness and illness has changed from the mid-20th century to modern times, moving from a diagnosis-focused to a person-focused definition of mental illnesses, and from an "absence of disease" model to one that stresses positive psychological function for mental health. Currently, wellness refers to the degree to which one feels positive and enthusiastic about oneself and life, whereas illness refers to the presence of disease. These definitions apply to physical as well as mental illness and wellness. In this article, we build on the essential concepts of wellness and illness, discuss how these definitions have changed over time, and discuss their importance in the context of health reform and health care reform. *Health reform* refers to efforts focused on health, such as health promotion and the development of positive well-being. *Health care reform* refers to efforts focused on illness, such as treatment of disease and related rehabilitation efforts.

## Introduction

In 1948, the World Health Organization defined health as "a state of complete physical, mental, and social well-being and not merely the absence of disease or infirmity" (1). Recently, the mental health and well-being aspects of this definition have been discussed in US health care and public health. These concepts can be integrated into the national health reform and health care reform initiatives under discussion. These terms refer to different phenomena. *Health reform* refers to efforts focused on health *—* health promotion and development of positive well-being. *Health care reform* refers to efforts focused on illness *—* treatment of disease and rehabilitation efforts. These linkages between reform and health and illness guide our analysis.

Almost 30 years ago, *wellness* and *illness* were proposed to be not 2 ends of the same continuum, but 2 independent continua (2,3). In this model, wellness refers to the degree to which one feels positive and enthusiastic about life. It includes the capacity to manage one's feelings and related behaviors, including the realistic assessment of one's limitations, development of autonomy, and ability to cope effectively with stress (3). By contrast, illness refers to the presence or absence of disease. A regimen of care that takes into account the full person would address both wellness and illness.

A healthy outlook can reduce the intensity and duration of illnesses, creating the so-called mind-body interaction. The reverse is also true. On average, public mental health clients (people served through state mental health care systems) die 25 years younger than other Americans (4). Other research shows that depression and its associated symptoms are major risk factors for the development of coronary heart disease and death after an initial myocardial infarction because of noncompliance in medical therapy and rehabilitation, adverse health behaviors, metabolic changes involving biomarkers linked to atherosclerosis and cardiac function, and factors associated with well-being (5).

## Definitions of Mental Illness

Definitions of mental illnesses have changed over the last half-century. *Mental illness* refers to conditions that affect cognition, emotion, and behavior (eg, schizophrenia, depression, autism). Formal clinical definitions now include more information (ie, we have moved from a partial to a more holistic perspective and transitioned from a focus on disease to a focus on health). The informal response has fostered a parallel transition from a focus on the stigma of mental illnesses to the recognition that mental health is important to overall health.

In the 1960s and 1970s, a person with a mental illness was defined by diagnosis alone, and there were few broad classes of mental disorders. National statistical data were reported by diagnoses (eg, cases of schizophrenia and cases of depression). People with mental illness were commonly stigmatized and institutionalized. At the same time, deinstitutionalization had begun and was accelerating.

A major shift occurred in care practices during the 1980s and 1990s. The national approach to care for people with severe mental illnesses was failing to support their needs. Large numbers of people with severe mental illnesses had been released from state mental hospitals, but few community mental health services were available to serve them. The population of homeless mentally ill people was growing rapidly. New definitions were needed to identify people with the most severe mental illnesses and to create a framework for new national programs. The formal work of the National Institute of Mental Health (NIMH) showed that diagnosis alone was not sufficient, and the additional concepts of disability and duration were added. Disability referred to major limitations in personal activities, and duration referred to the duration of disability and had a minimum threshold of 1 year. These concepts informed a definition for people with "severe and persistent mental illnesses," which is still used in mental health (6).

Subsequently, these efforts were extended to include another population with mental illnesses associated with lesser disabilities, and duration was removed from the definition. Currently, the person is viewed as paramount; strengths are emphasized and weaknesses de-emphasized. Recovery and full community participation are the goals. Here, recovery is a life-long process in which a person with a mental illness strives to participate fully in community life, even in the presence of continuing symptoms and disabilities.

These recent definitions use the wellness model, in which health and disease are viewed as 2 separate dimensions. Recovery is the bridge between the 2 that builds on the strengths of health to address the weaknesses of disease. Because many people with mental illnesses also have physical disorders, a dual emphasis on mental and physical health is essential. These emphases will be very important for health reform and health care reform.

The primary manuals used by epidemiologists, health management officials, and clinicians for mental disease classification are the American Psychiatric Association's Diagnostic and Statistical Manual of Mental Disorders (DSM), now in its 4th version (7), and the World Health Organization's Manual of the International Statistical Classification of Diseases, Injuries, and Causes of Death (ICD), currently in its 10th edition (8). Previous versions of the DSM and ICD have not been fully congruent so that the same diagnoses are listed in both systems. However, practitioners and insurers increasingly need to be conversant with both systems, especially in light of the new evidence on interactions between physical and mental health. Thus, the DSM-V Task Force has been developing the next edition to more closely align it with the 11th edition of ICD.

## Response, Remission, and Relapse

Depression is an important marker condition because it frequently co-occurs with a range of substance use and physical disorders. The NIMH Collaborative Depression Study (CDS) defined the desirable clinical endpoints of remission and recovery among people with depression (9). *Response* refers to a clinically significant reduction in depressive symptoms, whereas *remission* refers to the virtual absence of depressive symptoms after a response. *Relapse* refers to a return of depressive symptoms after remission, and *recovery* refers to sustained remission, with or without concurrent treatment; a return of depressive symptoms after recovery is a *recurrence*. These concepts can be used to describe the dynamics of any mental illness.

Several recent studies have shown that even subthreshold or minor depression is often associated with disability and poor psychosocial functioning, and a potentially more severe course that requires treatment (10). If left untreated or inadequately treated, depression can be a source of unnecessary personal distress, prolonged family burden, and a substantial number of illnesses and deaths (11).

## Definitions of Mental Health

One of the most significant developments of recent decades has been the emergence of theoretically based, empirically validated assessments of *positive* *psychological functioning,* including a sense of well-being and hope*.* One precursor, beginning in the 1950s and known as the "social indicators movement," pertained to quality of life (12). Several landmark studies described quality of life with a focus on how it varied by demographic characteristics and whether it changed across time (13).

Following this societal perspective, psychologists increased interest in the topic of subjective well-being, delineated its component parts (eg, life satisfaction, ratings of happiness), and investigated the influences of judgmental and motivational processes (14,15). Others approached psychological functioning from humanistic, existential, and life-span developmental perspectives that emphasized growth, meaning, and personal capacity (16). These formulations evolved into 2 broad orientations for defining psychological well-being: 1 focused on happiness and the other on human potential (17-19).

Further impetus came from the positive psychology movement (20) and many products following from it (21). These products cover wide territories of psychological experience: compassion, control, creativity, love, optimism, resilience, spirituality. Some of these human strengths represent well-developed areas of scientific inquiry, and others point to new areas for future studies. Two types of research are needed: 1) population-level studies on the distribution of well-being across society strata and 2) studies showing that well-being affects morbidity, mortality, and intervening biological processes.

In the past decade, psychological well-being has been investigated in national studies using empirical indicators such as life satisfaction, purpose, personal growth, environmental mastery, self-acceptance, autonomy, and positive relationships (22). As these studies document, the absence of mental distress does not guarantee the presence of well-being (ie, as specified above, mental illness and mental health are independent dimensions). In addition, these studies have clarified that psychological well-being is not equitably distributed in American society — older adults and people lacking educational attainment report lower levels of purpose, mastery, and growth (19), although in some instances, ethnic and minority status confers protective factors relative to social determinants (23).

New research has probed the idea that positive mental health may influence physical health and biological functioning. A recent review (24) summarizes evidence showing that high positive affect (measured in terms of happiness, joy, contentment, and enthusiasm) is linked with lower morbidity, increased longevity, and reduced health symptoms. Positive emotional style was also associated with better endocrine function (lower levels of cortisol, epinephrine, norepinephrine) and better immune response (higher antibody production, greater resistance to illness) (25). Similar findings have been reported linking positive affect to lower inflammatory response and lower blood pressure. Indicators of well-being have also been linked to biology. Older women with higher levels of purpose in life, personal growth, and positive relationships had lower cardiovascular risk (lower glycosylated hemoglobin, lower weight, lower waist-hip ratios, higher HDL cholesterol) and better neuroendocrine regulation (lower salivary cortisol throughout the day) (26). Those with positive relationships and purpose in life had lower inflammatory factors (eg, interleukin 6 [IL-6] and its soluble receptor [sIL-6r]) (27). Psychological well-being has been linked with brain function and asymmetric activation of the prefrontal cortex (28) and with reduced amygdala activation to aversive stimuli, accompanied by greater activation of the ventral anterior cingulated cortex (29). These advances clarify how well-being can arise in neural function.

If positive mental health is linked with better biological regulation and improved neural response to negative stimuli, can well-being be promoted among those who do not naturally possess such life outlooks or suffer from mental illness? Fava (30) examined "well-being therapy," which involves keeping a focus (through daily diaries) on positive experience and learning how to elaborate and savor such experience. This treatment has been linked with improved remission profiles among those suffering from recurrent depression. Fava also showed that such improvement persisted over a 6-year follow-up period.

## Social Determinants of Mental Health and Illness

Mental health and mental illnesses can both cause and be influenced by positive or negative social determinants of health (described further by Primm et al in this issue) defined as "the specific features of and pathways by which societal conditions affect health and that potentially can be altered by informed action" (31). These determinants include income, housing, stress, early childhood experiences, social exclusion, occupation, education level, sanitation, social support, discrimination (eg, racism), and lack of access to resources. Mental health promotion must consider the broad-scale social factors that can interact with biological determinants of mental illnesses. Negative determinants are often disproportionately distributed among minority populations, placing them at greater risk for the development of mental and physical illness and related mortality (32).

## Discussion

Our views have clear implications for health reform and for health care reform. First, several studies show that mental health is frequently intertwined with physical health and social conditions; attempts to understand different diseases, develop interventions, and design health promotion strategies will be more effective if rooted in a dynamic and complex biopsychosocial model of disease and health. Second, recent studies show that higher levels of well-being are linked with better regulation of biological systems and adaptive neural response, and may serve as a protective influence on good physical health. Third, different approaches are required for different subpopulations. The subpopulation with serious mental illnesses comprises only approximately one-quarter of all adults with a mental illness each year. The subpopulation with other mental illnesses, 75% of all adults with a mental illness each year, also requires a care system that addresses its service needs. Further, at any one time, substantial numbers of a third subpopulation are suffering from subsyndromal states or nonspecific psychological distress. Developing interventions that support these different subpopulations may have implications for the prevention of diagnosable and impairing mental illnesses and physical illnesses, and for recovery. Fourth, recovery is an important process that bridges illness and wellness (33); it deserves greater attention in the future.

Unfortunately, most evidence-based interventions only address mental illnesses and are seen as the province of mental health specialists. Yet primary care providers oversee most mental health care for the general population; they are essential partners in addressing physical health issues for both public mental health clients and the general population with less disabling mental illness.

For those with mental illnesses, the major concern of health care reform is promoting illness care based on wellness and well-being; recovery is a key concept. The major focus of health reform should be to promote wellness and well-being. Linked approaches can improve overall health, delay onset of chronic diseases, and enable personal success in family, community, and work.

The Centers for Disease Control and Prevention (CDC) and the Substance Abuse and Mental Health Services Administration (SAMHSA) can provide joint leadership in implementing the needed interventions. CDC has expertise in approaches to wellness and well-being; SAMHSA, in recovery-oriented, strength-based care. We recommend that CDC and SAMHSA undertake joint work guided by the vision of delaying the onset and mitigating the effects of mental illnesses, and promoting positive mental and physical health.
